# Role of Some Natural Antioxidants in the Modulation of Some Proteins Expressions against Sodium Fluoride-Induced Renal Injury

**DOI:** 10.1155/2018/5614803

**Published:** 2018-06-28

**Authors:** Ahlam M. Alhusaini, Laila M. Faddah, Naglaa F. El Orabi, Iman H. Hasan

**Affiliations:** ^1^Department of Pharmacology and Toxicology, College of Pharmacy, King Saud University, Riyadh, Saudi Arabia; ^2^Department of Pharmacology and Toxicology, Suez Canal University, Ismailia, Egypt

## Abstract

**Background:**

The aim of the present work is to find the effects of N-acetylcysteine (NAC) and/or thymoquinone (THQ) in the protection against acute renal injury induced by sodium fluoride (NaF).

**Method:**

Rats were distributed into five groups: G1 was normal (control), G2 was intoxicated with 10mg/kg NaF i.p., G3 was treated with 10mg THQ /kg, G4 was treated with 20mg NAC /kg, and G5 was treated with a combination of THQ and NAC. The previous treatments were given daily along with NaF for four weeks orally.

**Result:**

Rats intoxicated with NaF showed a significant increase in serum urea, creatinine, uric acid, renal lipid peroxidation, nitric oxide, and TNF-*α* levels, whereas the activity of superoxide dismutase (SOD) and glutathione (GSH) level was reduced. The expressions of Toll-like receptor-4 (TLR4), Lipocalin, vascular adhesion molecule-1(VCAM-1), and BAX proteins were upregulated, whereas Bcl-2 and NF-E2-related factor 2 (Nrf2) proteins expressions were downregulated. DNA fragmentation was also amplified. Histological analysis revealed that NaF caused a destructive renal cortex in the form of the glomerular corpuscle, the obliterated proximal and distal convoluted tubules, vacuolization in tubular cells focal necrosis, and cell infiltration. THQ and NAC supplementation counteracted NaF-induced nephrotoxicity as reflected by the increase in renal GSH and SOD. THQ and NAC ameliorated all the altered proteins expressions, improved renal architecture, and declined DNA fragmentation.

**Conclusion:**

The role of oxidative stress in the enhancement of NaF toxicity suggested the renoprotective effects of NAC and THQ against the toxicity of fluoride via multiple mechanisms.

## 1. Introduction

Fluoride is a naturally occurring compound and is widely used in various fields, including pharmaceutical preparations, agriculture, pesticides, and surfactants [1]. In pharmacological doses, fluoride is widely used due to its beneficial effects for teeth and bone development; accordingly, in many countries, it has been added to drinking water sources [2]. However, the uncontrolled consumption of fluoride, accidental or suicidal, by human and experimental animals was shown to be toxic to many organs such as kidneys, gonads, and nerve cells [3] and may cause many side effects including dental and skeletal fluorosis, infertility, and mental retardation [4–6]. Fluoride toxicity is closely linked with a huge number of toxic effects on cellular metabolism, such as oxidative stress (OS), suppression of protein synthesis, alteration in the gene expression, and DNA damage [7]. The ability of fluoride to initiate apoptosis in different organs has been documented, for instance, kidney, [7] livers, [8] brains [9], and lung [10].

Dramatically, the number of population suffering from element toxicity is growing swiftly with a significant clinical and economic burden. Therefore, fluoride toxicity requires urgent and effective therapeutic interventions to compensate tissue damage which is mainly caused by liberation of free radicals. This concept has opened a new area for using the antioxidants in the prevention and therapy of many diseases. Consequently, there is an urgent need to use natural products for many diseases remedy due to their availability and fewer side effects. Herein, we will use N-acetylcysteine (NAC) [11] and thymoquinone (THQ) [12] as natural antioxidants to investigate their therapeutic role in the amelioration of renal toxicity induced by sodium fluoride (NaF).

Clinically, NAC is used as an antioxidant precursor of glutathione (GSH) for the treatment of paracetamol toxicity [13]. Furthermore, NAC has some beneficial effects in psychiatric disorders [11].

THQ is the bioactive constituent of black seed essential oil [14]. It has many pharmacological properties including antioxidant, anti-inflammatory, chemoprotective, and antiproliferative activities [15]. It is well established that THQ functionally acts by modifying the physiological and biochemical processes involved in ROS generation [12]. THQ activates SOD and glutathione transferase, increases GSH levels, and suppresses lipid peroxidation [12].

The present study is the first to explore the renoprotective effects of NAC and THQ alone or in combination against NaF-induced kidney injury and to explore novel molecular pathways implicated in their mechanisms of action, as well as their role in the suppression in the DNA damage.

## 2. Materials and Methods

### 2.1. Chemicals

All chemicals used were from Sigma Company (Sigma, St. Louis, MO, USA). Commercial kits for the assay of urea, uric acid, and creatinine were obtained from Randox Company. Primary and secondary antibodies for TLR4, Lipocalin, VCAM, BAX, Bcl-2, and Nrf2 were obtained from Santa Cruz Biotechnology (CA, USA).

Male albino rats (80–190 g) were supplied and animal utilization protocols were performed in accordance with the guidelines provided by the Experimental Animal Laboratory and approved by the Animal Care and Use Committee. The animals were supplied by the College of Pharmacy at King Saud University (Riyadh, Saudi Arabia) and were provided free access to standard laboratory diet of known composition and water ad libitum. The rats were maintained at a normal atmospheric temperature (23 ± 2°C) on a 12 h light/dark cycle.

Experimental design: Fifty rats were distributed into five groups of ten rats each; they were treated as follows: G1 was normal untreated (control) group, G2 was intoxicated with 10 mg/kg/day NaF (i.p.) [16], G3 was treated with THQ at a dose of 10 mg/kg/day [17], G4 was treated with 20 mg/kg/day of NAC [18], and G5 was treated with THQ and NAC. The antioxidants in question were given orally after two hours from NaF injection for four weeks.

Rats were sacrificed after the end of the experiment and sera were separated from blood by centrifugation at 3000 rpm for 20 min. Thereafter, parts of six right kidneys were homogenized in phosphate buffer (20% homogenates). After centrifugation of the homogenates, the supernatants were kept at -80°C. Other parts of the six right kidneys were frozen under liquid nitrogen for Western blotting. Three right kidneys were kept in 4% formalin for histopathological examination.

### 2.2. Experimental Animals

#### 2.2.1. Biochemical Serum Analysis

Serum analysis: Serum creatinine, urea, and uric acid were analyzed using kits (Randox Company), whereas serum TNF-*α* level was measured using a high sensitive rat ELISA kit (Immuno-Biological Laboratories Co., Ltd. Takasaki-Shi, Gunma, 370-0831, JAPAN).

Kidney tissues analysis: MDA, GSH, and SOD were determined according to the methods of Uchiyama and Mihara (1978) [19], Ellman (1959) [20], and Marklund and Marklund (1974) [21], respectively. NO (total nitrite) in the kidney homogenates was determined as nitrite content using Griess reagent [22].

#### 2.2.2. Determination of DNA Fragmentation

DNA samples were fragmented by ultrasound using Covaris S220 (Covaris, Woburn, MA, USA) [23].

#### 2.2.3. Histological Analysis

Thinly sliced renal sections (5 *μ*m) were stained with Haematoxylin and Eosin (H&E) and examined under a microscope.

#### 2.2.4. Western Blot Analysis

Western blot analysis was used to determine the protein levels of Toll-like receptor-4 (TLR4), Lipocalin, vascular adhesion molecule-1 (VCAM-1), Bcl-2-associated protein X (BAX), B-cell lymphoma/leukemia-2 (Bcl-2), and NF-E2-related factor 2 (Nrf2) in kidney homogenate and was performed as described previously [24]. Briefly, samples containing 60 *μ*g of protein were mixed with an equal volume of loading buffer (2 % SDS, 25 % glycerol, 10% 2-mercaptoethanol, 0.01% bromophenol blue, and 62.5 mM Tris HCl, pH 6.8). Samples then were denatured by incubation in thermomixer at 99°C for 5 min. Denatured samples with the protein marker were separated on sodium dodecyl sulfate-polyacrylamide gel electrophoresis (SDS-PAGE). Samples were electrophoresed at 60 V for 30 minutes followed by 120 V for approximately 90 minutes in running buffer (25 mM Tris, 192 mM glycine, and 0.1% SDS, pH 8.6). Molecular weights of the different proteins were estimated using protein markers of known molecular weight (Bio-Rad Laboratories, Hercules, CA, USA). The separated proteins were electrophoretically transferred to polyvinylidene difluoride (PVDF) membranes in a semidry system containing transfer buffer (25 mM Tris, 192 mM Glycine, 20% (v/v) methanol, pH ~8.3). Protein blots were blocked overnight at 4°C in a solution containing 5% skim milk powder, 2% BSA, and 0.5% Tween-20 in Tris-buffered saline (TBS) solution (0.15 M NaCl, 3 mM KCl, 25 mM Tris-base). Thereafter, the blocking solution was removed and the blots were rinsed three times (5 minutes) in a wash buffer (0.1% Tween-20 in TBS).

Subsequently, membranes were incubated overnight at 4°C with a primary antibody: anti-TLR-4 (SAB1301541), anti-Lipocalin (AF1857), VCAM-1 (ab134047), Bcl-2 (ab692), BAX (ab53154), NF-kB (ab32536), Nrf-2 (ab137550), and anti-beta-actin rabbit polyclonal antibody. The primary antibody solution was removed by washing three times in TBST. Blots were then incubated for one hour with horseradish peroxidase- (HRP-) conjugated goat anti-mouse (1:10000) secondary antibody and goat anti-rabbit secondary antibody (1:10000) diluted in TBST buffer. The excess secondary antibodies were removed by washing three times in TBST for 5 minutes. Finally, blots were developed using ECL detection reagents for 2 minutes prior to image acquisition. The protein bands were visualized by Image Quant LAS 4000 mini (GE Health Care, UK) and semiquantified by using ImageJ version 1.45 software. The TLR4, Lipocalin, VCAM-1, BAX, Bcl-2, NF-kB, and Nrf2 protein levels were normalized against the loading control (beta-actin) by dividing the value of the target protein by the value of the beta-actin. The relative values were normalized to the control whose value is fixed arbitrarily to one and assigned as a fold of induction.

### 2.3. Statistical Analysis

Statistical comparison between groups was performed using one-way analysis of variance (ANOVA) followed by Tukey-Kramer multiple comparison test. The results were considered as significant when p<0.05. Statistical tests were conducted using GraphPad Prism 5.00 (GraphPad Prism, San Diego, California, USA) and SPSS 21 (IBM, USA).

## 3. Results

Herein, NaF significantly elevated serum creatinine, urea, and uric acid levels compared to control (p≤0.01, p≤0.001, p≤0.01), respectively (Table 1). THQ solo or in combination with NAC has effectively declined the previous serum biochemical parameters (Table 1). Moreover, serum TNF-*α* level was increased upon NaF intoxication and declined by using the antioxidants in question supplementation (Table 1).

**Figure 1:**
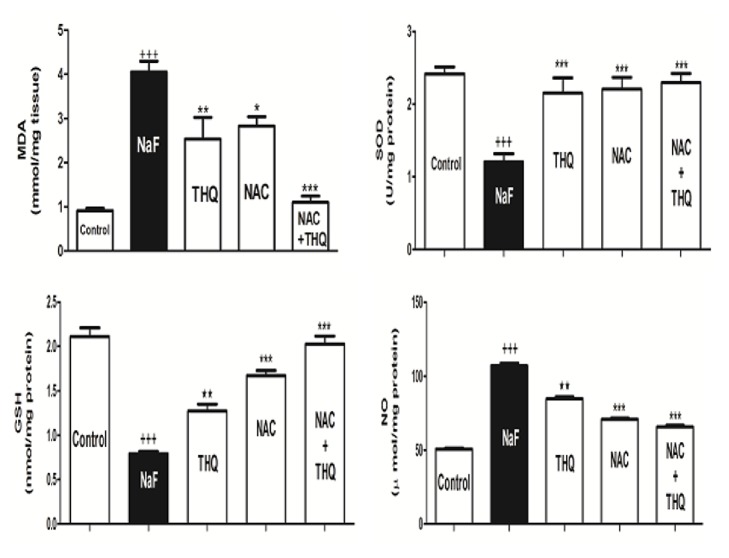
Oxidative stress and antioxidant biomarkers (MDA, NO, GSH, and SOD) in renal tissues of rats in control, NaF-intoxicated, and all treated groups. MDA: malondialdehyde; NO: nitric oxide; GSH: glutathione; SOD: superoxide dismutase; NaF: sodium fluoride; NAC: N-acetylcysteine; THQ: thymoquinone. Notes: Data are presented as mean ± SEM (N=6). ^+++^p ≤ 0.001 versus control, and ^*∗∗∗*^p ≤ 0.001 versus NaF-intoxicated group.

Intoxication with NaF produced a significant increase in MDA and NO in renal tissue (p<0.001) with a concomitant depletion of renal SOD (p<0.001) and GSH content (Figure 1). THQ and NAC treatments improved the changes in the earlier measured parameters (Figure 1). The combined treatment of both NAC and THQ significantly reduced MDA (p<0.001) (Figure 1). THQ and/or NAC produced a high significant increase in the SOD and GSH levels compared to NaF-intoxicated group (p<0.001).

**Figure 2:**
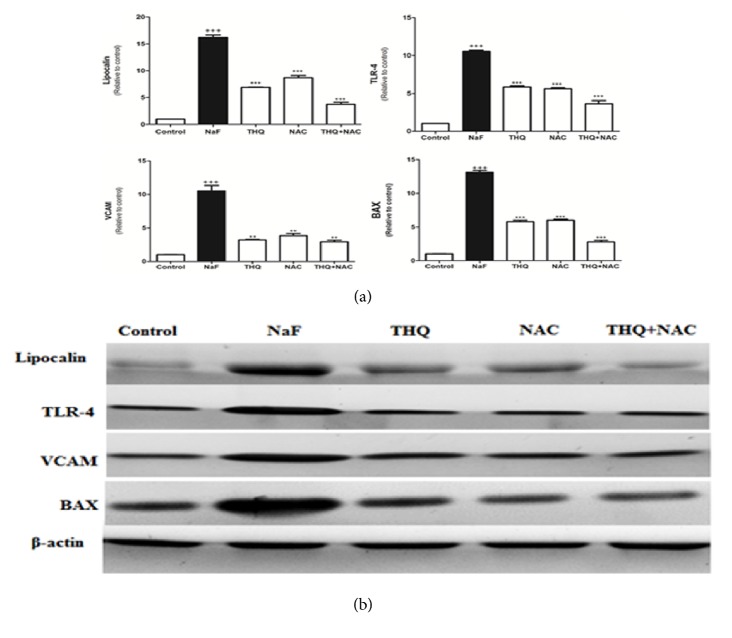
(a) The densitometry analysis of the expression of Lipocalin, TLR-4, VCAM, and BAX proteins in control, NaF-intoxicated, and all treated groups. (b) Western blot analysis of the expression of Lipocalin, TLR-4, VCAM, and BAX proteins in control, NaF-intoxicated, and all treated groups. (Data corrected by *β*-actin and expressed as protein / *β*-actin.) NaF: sodium fluoride; NAC: N-acetylcysteine; THQ: thymoquinone; TLR-4: Toll-like receptor-4; VCAM: vascular adhesion molecule; BAX: Bcl-2-associated protein X. Notes: Data are presented as mean ± SEM (N=6). ^+++^p≤ 0.001 versus control, and ^*∗∗∗*^p≤ 0.001 versus NaF-intoxicated group.

**Figure 3:**
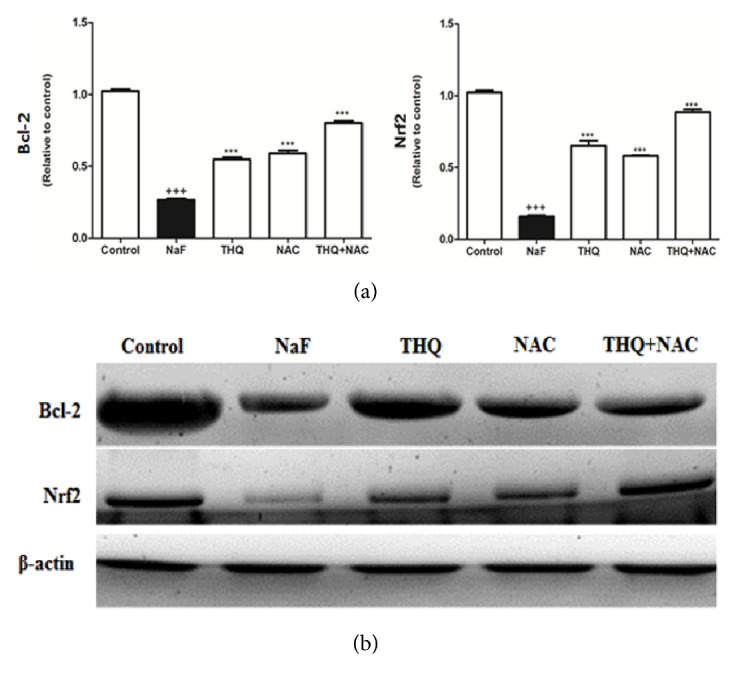
(a) The densitometry analysis of the expression of Bcl-2 and Nrf2 proteins in control, NaF-intoxicated, and all treated groups. (b) Western blot analysis of the expression of Bcl-2 and Nrf2 proteins in control, NaF-intoxicated, and all treated groups. NaF: sodium fluoride; NAC: N-acetylcysteine; THQ: thymoquinone; Bcl-2: B-cell lymphoma/leukemia-2; Nrf2: NF-E2-related factor 2. Notes: Data are presented as mean ± SEM (N=6). ^+++^p≤ 0.001 versus control, and ^*∗∗∗*^p≤ 0.001 versus NaF-intoxicated group.

**Figure 4:**
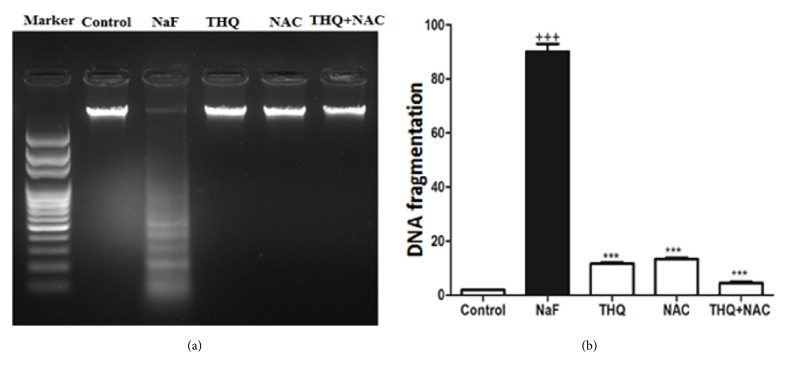
(a) Intoxication with NaF caused a significant increase in the formation of DNA fragmentation, while the use of antioxidants decreased the DNA degradation (the DNA was electrophoresed using agarose gel). (b) Quantitative analysis of DNA fragmentation levels in control, NaF-intoxicated, and all treated groups. NaF: sodium fluoride; NAC: N-acetylcysteine; THQ: thymoquinone. Notes: Data are presented as mean ± SEM (N=6). ^+++^p≤ 0.001 versus control, and ^*∗∗∗*^p≤ 0.001 versus NaF-intoxicated group.

Protein expressions of Lipocalin, TLR-4, VCAM, and BAX were upregulated upon NaF treatment (Figures 2(a) and 2(b)) whereas there was a decline of protein expression of Bcl-2 and Nrf2 in the NaF-intoxicated group compared to the control (p<0.001) (Figures 3(a) and 3(b)). Treatment with the antioxidants ameliorated all the altered protein expressions. DNA fragmentation was significantly increased by NaF intoxication compared to the control group (p<0.001), while treatment with the aforementioned antioxidants attenuated DNA degradation (Figures 4(a) and 4(b)).

**Figure 5:**
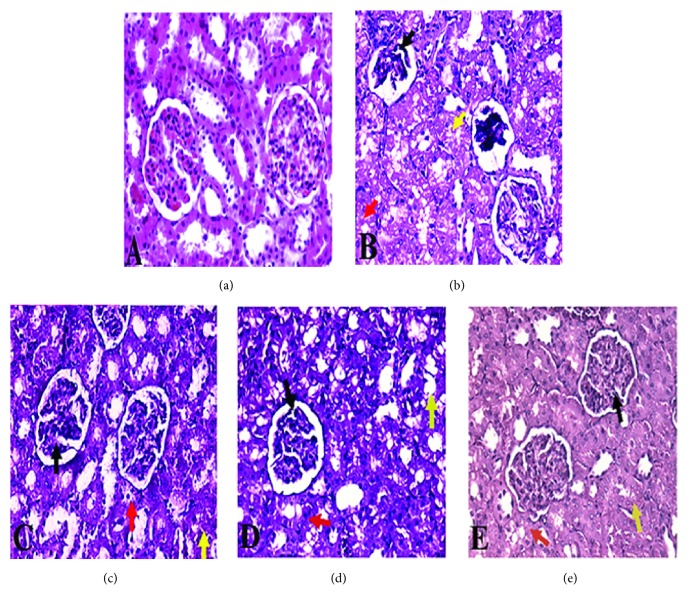
Histological analysis of kidneys' sections (scale= x400) from (a) normal architecture of control group; (b) NaF-intoxicated group showing damaged and obliterated glomerular corpuscle (black arrow), proximal (yellow arrow), and distal (red arrow) convoluted tubules; (c) and (d) kidneys' sections from NAC-treated group and THQ-treated group, respectively, showing renal cortex in the form of normal renal corpuscle with normal glomerulus (black arrow), normal pattern of proximal convoluted tubules (red arrow), and few distal convoluted tubules lined with mildly destructed epithelium, mild vacuolization in tubular epithelium (yellow arrow); and (e) kidney section from combined-treated group showing renal cortex in the form of almost normal renal corpuscle with normal glomerulus (black arrow), few proximal convoluted tubules showing destructed epithelial lining, vacuolization in tubular cells focal necrosis, and cell infiltration (red arrow) and distal convoluted tubules lined by a lower, cuboidal epithelium (yellow arrow). (Scale 400.)

The histological study of kidney sections from of NaF-intoxicated group showed a destructive renal cortex in the form of the glomerular corpuscle, the obliterated proximal and distal convoluted tubules, vacuolization in tubular cells focal necrosis, and cell infiltration (Figures 5(a) and 5(b)). In contrast, kidney sections from groups treated with NAC and/or THQ showed normal renal cortex with normal glomerulus; the normal pattern of proximal convoluted tubules is lined by a thick columnar epithelium and distal convoluted tubules are lined by a lower, cuboidal epithelium (Figures 5(c), 5(d), and 5(e)). Interestingly, the concomitant treatment of NAC with THQ was the most protective one against NaF intoxication (Figure 5(e)).

## 4. Discussion

Fluoride is abundant natural substance and prevalent industrial pollutant that is detected in virtually all environmental matrices [25]. Fluoride has high cariostatic capacity that makes it extensively considered in preventive dentistry. It has been progressively supplied to unconventional deliverance such as toothpaste and mouth wash, fluoridated water supplies, and food products. The extensive utilization of these fluoridated products provokes serious health problem [26].

Although the mainly noticeable early toxic effects of fluoride are dental and skeletal fluorosis, which are prevalent in place with prominent exposure to fluoride, it is also able to cross the cell membranes, go through soft tissues, and hence alter tissue metabolism and functions [27]. Fluoride initiated excessive generation of ROS, lipid peroxidation, and NO levels and depletion of GSH and SOD contents and ATP level [27–29].

It was documented that renal function is greatly affected by NaF toxicity, as kidneys are the principal organs involved the excretion and retention of NaF [30]. NaF intoxication induced a significant elevation in the kidney biochemical markers including urea, uric acid, creatinine, and phosphate levels and decrease in the levels of calcium [31].

Fluoride's nephrotoxicity is mainly dependent on diffusion of fluoride ions in the form of hydrogen fluoride. Fluoride is freely filtered through glomerular capillaries and then experiences a variable degree of tubular reabsorption. NaF causes impairment to the mitochondrial function, consequently suppressing cellular respiration, and initiates free radicals production leading to renal dysfunction [32, 33].

Few studies were performed on antioxidant therapies and their mechanism on kidney health, so there is an urgent need for investigation of the use of antioxidants in the treatment of fluoride-induced nephrotoxicity. The current study revealed the renoprotective potential of NAC and THQ either alone or in combination with kidney injury induced by NaF.

Herein, using of NAC and/or THQ downregulated the elevated kidney biomarkers as well as lipid peroxide and NO induced by NaF toxicity. Moreover, the treatment with these natural antioxidants increased the activity of SOD and reduced GSH level. OS is a well-known mode of action of fluoride exposure that has been observed either in vitro or in vivo. Scavengers of ROS, as NAC and THQ, restore the intracellular redox homeostasis and lipid peroxidation, thereby restoring the altered serum renal biomarkers [34–36].

It was previously reported that treatment of normal subjects with either NAC or THQ conserves levels of GSH level and other antioxidants enzymes to their normal values [37, 38].

The molecular mechanism of the pathogenesis of NaF toxicity is poorly understood [3], so this study is focused on exploring different mechanisms involved in the pathogenic effect of NaF. TLRs play a fundamental role in the innate immune system by triggering proinflammatory signaling pathways and promote the activation of leukocytes [39]. In the kidney, TLRs are widely expressed in tubular epithelial cells, mesangial cells, endothelial cells, and infiltrating macrophages [40]. It has been proved that, during renal fibrosis, TLRs were activated in these cells [41]. Tubular epithelial cells are among the nonimmune cells that express TLR1, 2, 3, 4, and 6, proposing that these receptors might be involved in the activation of immune responses in tubule-interstitial injury [40]. TLRs are involved in most of the renal inflammation [41]. In the present study, TLR-4 protein expression is upregulated upon NaF treatment while NAC and THQ administration alone or in combination downregulated its expression.

Lipocalins (LCNs) constitute a family of small secreted proteins and act in regulating the activity of immune system. Many LCNs take action as transporters for specific proteins and fatty acids, and LCN2-iron complexes bind to cell surface receptors to carry iron into the cell. The sequestration of iron by LCN2 could play a protective role in the innate immune response against bacterial infections and may be involved in the initiation of apoptosis. It has been recognized that the amount of urinary glycoprotein LCN2 increases after kidney injury due to the reduction of reabsorption by the proximal tubules or direct secretion from injured tissues [42]. LCN2 gene expression is upregulated in a diversity of cells in acute situations, including cancer, acute liver or kidney injury and infection [42]. As shown in the current study, LCN protein expression is enhanced due to NaF intoxication, whereas NAC and/or THQ administration caused a reduction in its expression.

Herein, NaF increased in TNF-*α* level compared with normal group whereas the treatment with the antioxidants significantly decreased its level compared to the NaF-intoxicated group. Stimulation of leukocyte adhesion to the endothelium by TNF-*α* is mediated by the upregulation of adhesion molecules on the endothelial cell surface [43]. TNF-*α* enhances the expression of intracellular adhesion molecule-1 (ICAM-1), VCAM-1, and E-selectin expression in many organs, such as kidney, liver, and lung. Subsequently, TNF-*α* receptor mediates the stimulation of VCAM-1 and ICAM-1 expression and is critically involved in the control of leukocyte organ infiltration. It was documented that NaF induced an elevation of the inflammatory marker TNF-*α* [44].

BAX is well-known apoptotic agent that enhances mitochondrial channels with the release of endonuclease G (apoptotic protein) [45]. It was reported by Xu and colleagues that NaF enhances apoptosis in renal tubules through activation of BAX and reduction of Bcl-2 level [46]. Other researchers reported that NaF exhibits kidney apoptosis by caspase-mediated pathway, and DNA damage could be related to this process [30].

Nrf2 is a transcription factor that is important for the antioxidant responsive element- (ARE-) mediated antioxidant genes which is a major pathway regulating detoxifying and antioxidant enzymes under conditions of oxidative or electrophilic stress. It was found that Nrf2 was downregulated by fluoride treatment [46].

Herein, our results were matched with the previous data which showed that NaF induced apoptosis represented by the upregulation of BAX (proapoptotic) together with DNA fragmentation and downregulation of Bcl-2 (antiapoptotic) and Nrf2 protein expression. BAX/Bcl-2 ratio was also increased as well. Treatment with the antioxidants in question was effective against fluoride-induced renal injury through their anti-inflammatory effect via downregulating the TNF-*α* and also prevents the apoptotic events via upregulating the expression of Bcl-2 and Nrf2 and the suppression of BAX, TLR4, Lipocalin, and VCAM expressions. Accordingly, the outcome of the present study evidently proved that THQ and NAC combination attenuates NaF-induced oxidative stress-mediated renal toxicity and it is the first time to ensure the involvement of Bcl-2, BAX, Nrf2, TLR4, Lipocalin, and VCAM proteins' expressions in both NaF toxicity and treatments. The combination regimen is considered as an efficient candidate for the treatment of NaF-induced renal toxicity.

## Figures and Tables

**Table 1:** Serum levels of kidney function parameters, BAX/Bcl-2 ratio, and renal TNF-*α* in control, NaF, and all treated groups.

Parameters	Control	NaF	THQ	NAC	THQ + NAC
Creatinine (mg/dl)	1.4± 0.21	2.2± 0.16^++^	1.6± 0.082^*∗*^	1.8± 0.068^*∗*^	1.5± 0.19^*∗*^

Urea (mg/dl)	16± 1.2	80± 3.7^+++^	36± 1.9^*∗∗∗*^	27± 1.8^*∗∗∗*^	23± 1.7^*∗∗∗*^

Uric Acid (mg/dl)	3.7±0.41	7.3±0.75^++^	6.9±0.75^*∗*^	5.7±1.0^*∗*^	4.5±0.39^*∗∗*^

BAX/Bcl-2 ratio	100.5±0.34	5065±45.2^+++^	1143±37.3^*∗∗∗*^	1053±9.23^*∗∗∗*^	325.4±20.9^*∗∗∗*^

TNF-*α* (pg/ml)	34.65±1.04	203.8±6.58^+++^	87.63±3.54^*∗∗∗*^	105.1±1.9^*∗∗*^	65.26±3.17^*∗∗∗*^

Notes. Data are shown as mean ± SEM (N=6). ^+++^P≤ 0.001 versus control and ^*∗∗∗*^P≤ 0.001 versus NaF-treated group.

## Abbreviations

TLR-4: Toll-like receptor-4
LCN: Lipocalin
VCAM-1: Vascular adhesion molecule-1
Bcl-2: B-cell lymphoma/leukemia-2
BAX: Bcl-2-associated protein X
Nrf2: NF-E2-related factor 2
GSH: Glutathione
SOD: Superoxide dismutase
NAC: N-acetylcysteine
THQ: Thymoquinone.
